# Commentary: The Long History of Vitamin C: From Prevention of the Common Cold to Potential Aid in the Treatment of COVID-19

**DOI:** 10.3389/fimmu.2021.659001

**Published:** 2021-04-01

**Authors:** Harri Hemilä, Elizabeth Chalker

**Affiliations:** ^1^ Department of Public Health, University of Helsinki, Helsinki, Finland; ^2^ School of Public Health, University of Sydney, Sydney, NSW, Australia

**Keywords:** ascorbic acid, common cold, coronavirus, immune system, pneumonia, respiratory tract infections, rhinovirus

## Introduction

A review of the effects of vitamin C on the immune system and respiratory tract infections was recently published (1). We are the authors of a review of vitamin C and the common cold (2), which was cited in the article. We consider that some of the authors’ statements are inaccurate and here we describe the issues on which we disagree.

## Statements on Vitamin C and the Common Cold

Cerullo stated “According to Pauling, a daily vitamin C intake of 1,000 mg can reduce the incidence of colds by about 45% (3, 4). However, other clinical studies with similar aims failed to demonstrate its efficacy [(5–8); references renumbered]”. The cited references do not support the last statement.

The large Anderson (1972) trial (5) did not fail to demonstrate the efficacy of vitamin C. In contrast, the abstract summarizes the findings as follows *“there was a statistically significant difference (P <0.05) between the two groups in the number of subjects who remained free of illness throughout the study period. Furthermore the subjects receiving the vitamin experienced approximately 30% fewer total days of disability (confined to the house or off work) than those receiving the placebo, and this difference was statistically highly significant (P <0.001)”* (5); see **Table 1**. This does not support the claimed inefficacy of vitamin C.

**Table 1:** Effect of vitamin C on the proportion of participants with illness in the large Anderson (5) trial.

Outcome	Intervention		Effect of vitamin C (95% CI)	P (2-tail)
	Vitamin C	Placebo		
No days confined indoors	232/407 (57%)	195/411 (47%)	9.6 (2.7, 17) pp	0.006
No days off work	275/407 (67%	243/411 (59%)	8.4 (1.8, 15) pp	0.012
No days with nose or throat symptoms	131/407 (32%)	101/411 (25%)	7.6 (1.4, 14) pp	0.016
Free of any illness during the trial	105/407 (26%)	76/411 (18%)	7.3 (1.6, 13) pp	0.012

Participants were administered 1 g/day vitamin C regularly and 3 g/day extra for three days when they were sick. The 9.6 percentage point difference in the proportion of participants who had no days confined indoors indicates that 9.6% of participants benefited from vitamin C on the basis of this outcome in the particular context of the Anderson (5) trial. Anderson restricted to persons who normally experienced at least one cold in the period January to March. The data for “days confined indoors” is from p. 506 and Table VI, the data for “days off work” is from p. 506, the data for the number of subjects who did not experience any nose or throat symptoms are from p. 508, and the data for any illness is from p. 505 of (5). An earlier version of this table was published as Table 20 in ref. (9). The calculations were done with the prop.test program of the R package.

pp, percentage point difference in the outcome between the two groups.

Karlowski et al. (1975) stated *“the effects [of vitamin C] demonstrated might be explained equally well by a break in the double blind”* (6). However, Hemilä showed that the statistical analysis in the Karlowski trial was flawed. The reanalysis concluded that *“The most important conclusions from Karlowski’s study are that therapeutic vitamin C supplementation during a common cold episode appears to be as effective as regular supplementation, and that there appears to be linear dose dependency at least up to 6 g/day. These findings suggest that large therapeutic vitamin C doses might alleviate the symptoms of the common cold substantially”* (9–11).

The Chalmers meta-analysis (1975) calculated that vitamin C shortens common cold duration just by 0.11 (0.24 SE) days (7). However, there were numerous errors in Chalmers’ calculations which when corrected led to an estimated reduction in cold duration of 0.93 (0.22 SE) days (9, 12, 13). This finding is significant statistically and clinically.

The Dykes and Meier review (1975) (8), also contained substantial errors as detailed previously (9, 13).

Cerullo also writes “Vorilhon et al. (14), analyzed eight RCTs and confirmed that vitamin C supplementation … is not effective, compared to placebo, in reducing the incidence of upper respiratory tract infections (URTI) in 3,135 children…, although the administration can reduce the duration of URTI by 14%”. However, we showed that the Vorilhon meta-analysis contained errors (15). Thus, the quoted figures are not correct.

Cerullo further writes “Kim et al. (16) carried out a large randomized, double-blind, placebo-controlled trial in 1,444 Korean soldiers, 695 of whom received vitamin C (6 g/day) for 30 days. They showed that the vitamin C group had a 0.80-fold lower risk of getting the common cold compared to the placebo group (n = 749).” The “0.80-fold lower risk” implies that there was a 20% reduction in the risk, however, 0.80 is the odds ratio which is inappropriate for common outcomes such as the common cold (17). The correct risk reduction for catching a cold was just 8.4% (18). Further problems are described elsewhere (18).

## Statements on Vitamin C and Other Medical Conditions

The review by Cerullo et al. is also misleading on issues other than the common cold. With respect to vitamin C and pneumonia, they cite the Padhani (2020) meta-analysis (19) as follows *“the most recent meta-analysis including 2,774 participants from seven clinical studies, underlined that current evidence is insufficient to sustain the efficacy of vitamin C supplementation in preventing or treating pneumonia, due to the small number of trials and very low quality of the existing results* (19)*.”*

The Padhani review also contains significant errors. We described several of these including the inclusion and exclusion of trials and the statistical calculations (20).

As to the effect of vitamin C on the mortality of ICU patients, Cerullo referred to the CITRIS-ALI trial as follows: “findings from the CITRIS-ALI study (21) showed a reduced mortality at day 28 in the vitamin C group (29.8%) compared to the placebo group (46.3%)”. Although the figures are correct, the CITRIS-ALI trial authors flagged concerns with multiple comparisons, which is not mentioned by Cerullo. That said, the multiple comparisons interpretation has been challenged (22). Furthermore, there was a significant difference between the vitamin C and placebo groups during the 4-day vitamin C supplementation period as mortality was decreased by 81% (95% CI 45% to 94%), whereas no difference between trial groups was seen after vitamin C administration was discontinued (22).

## Discussion

Our meta-analysis on vitamin C and the common cold found that vitamin C did not decrease the average number of colds in general community trials (2). However, we found that in five trials with 598 physically active participants vitamin C decreased common cold risk by 52% (P < 0.00001) (2), yet this effect was not mentioned by Cerullo (1). Furthermore, in one of the largest trials to date, Anderson (1972) (5) found that the occurrence of outcomes *“not confined to the house”*, *“not off work”*, and *“not ill during the trial”* because of common cold related symptoms were all about 8 percentage points lower in the vitamin C group than in the placebo group (**Table 1**). This means that 1 in every 12 participants in the trial benefited from vitamin C.

Vitamin C is known to cure scurvy, but it may also have an effect on other conditions (2, 4, 5, 9–13, 21, 22). We consider that some parts of the Cerullo review did not adequately describe the reported effects of the vitamin. There has been much discussion over decades about vitamin C, unfortunately much of it erroneous. Solid evidence is required to determine whether vitamin C, which is cheap and safe, is effective against other conditions, without the bias that has often been caused by self-perpetuating errors.

## Author Contributions

All authors contributed to the article and approved the submitted version.

## Conflict of Interest

The authors declare that the research was conducted in the absence of any commercial or financial relationships that could be construed as a potential conflict of interest.
